# Supplementary material for: A conserved motif suggests a common origin for a group of proteins involved in the cell division of Gram-positive bacteria
Source: PLoS One. 2023 Jan 20;18(1):e0273136. doi: 10.1371/journal.pone.0273136 (PMC9858780; doi:10.1371/journal.pone.0273136)
Supplement: S1 File — Furthermore, the following data are available at https://data.mendeley.com/datasets/bn627zbymx: (i) a cluster-map file, which can be navigated interactively in CLANS and gives direct access to all the sequences in this study, (ii) the sequence annotations for representatives of DivIVA-like proteins one for each cartoon shown in Fig 2B–2D, and (iii) the sequences and structural models of the PolyDIVs modeled with AlphaFold, both natural and artificial. (DOCX) [file pone.0273136.s001.docx]

**Supplementary Information: A conserved motif suggests a common origin for a group of proteins involved in the cell division of Gram+ bacteria**

Mikel Martinez-Goikoetxea, Andrei N. Lupas*

Department of Protein Evolution, Max Planck Institute for Developmental Biology, 72076 Tübingen, Germany

* To whom correspondence should be addressed:

Andrei N. Lupas

Tel.: +49 7071 601 341

Fax: +49 7071 601 349

**E-mail:** [**andrei.lupas@tuebingen.mpg.de**](mailto:andrei.lupas@tuebingen.mpg.de)

**
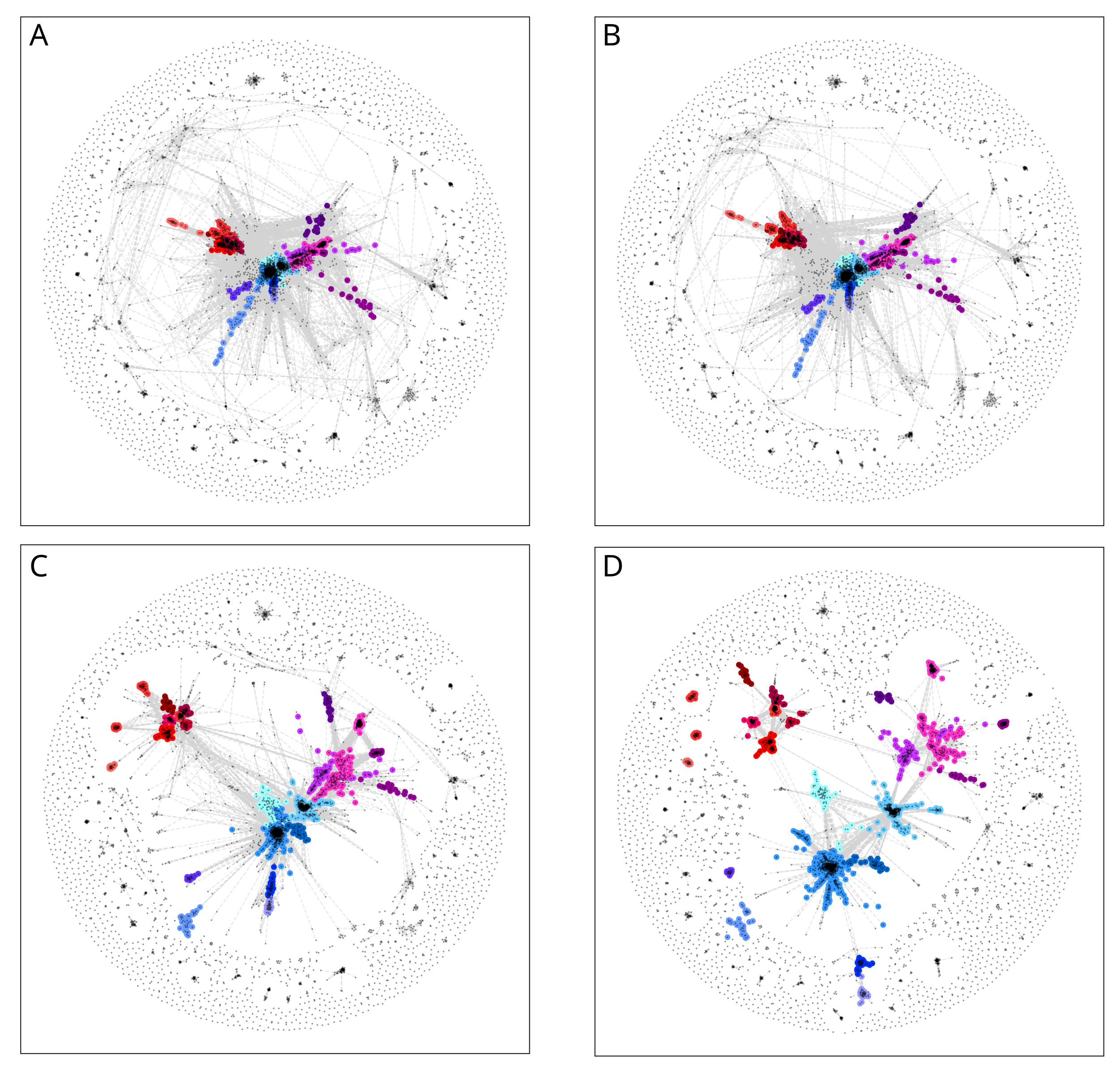
**

**S1 Figure. Series of CLANS maps at different P-value thresholds.**

Shown are the CLANS maps, colored to match Fig. 2A in the main manuscript, at the P-value thresholds of (A) p=1, (B) p=1E-5, (C) p=1E-10, (D) p = 1E-15. As the value is decreased, only the most significant matches are taken into account to construct the map, and thus the sub-groups are revealed

**S2 Appendix. Evidence of continuous genetic duplication events**

While performing BLAST searches of DivIVA-like domains against the proteomes of the seed database ([https://pubseed.theseed.org](https://pubseed.theseed.org/)), we found instances of non-streptomyces actinobacteria that featured several additional DivIVA-like proteins besides one copy of DivIVA and one of FilP. For example, in Pseudonocardia dioxanivorans CB1190, we found two sequences (seed identifiers 675635.11.peg. 1714 and 1181) that by BLAST searches against our cluster map could be reliably identified as FilP-like proteins. In Actinoplanes sp. SE50/110, we found two genomically adjacent FilP-like sequences (134676.3.peg. 7340 and 7341), and two sequences that closely resembled DivIVA (134676.3.peg. 2009, 1670). Finally, by the manual analysis of the GCsnap results, we also found that Nocardiopsis chromatogenes has two genomically adjacent proteins (WP_017626108.1, WP_017626109.1) that by BLAST searches can be mapped to different clusters within the PolyDIV group.


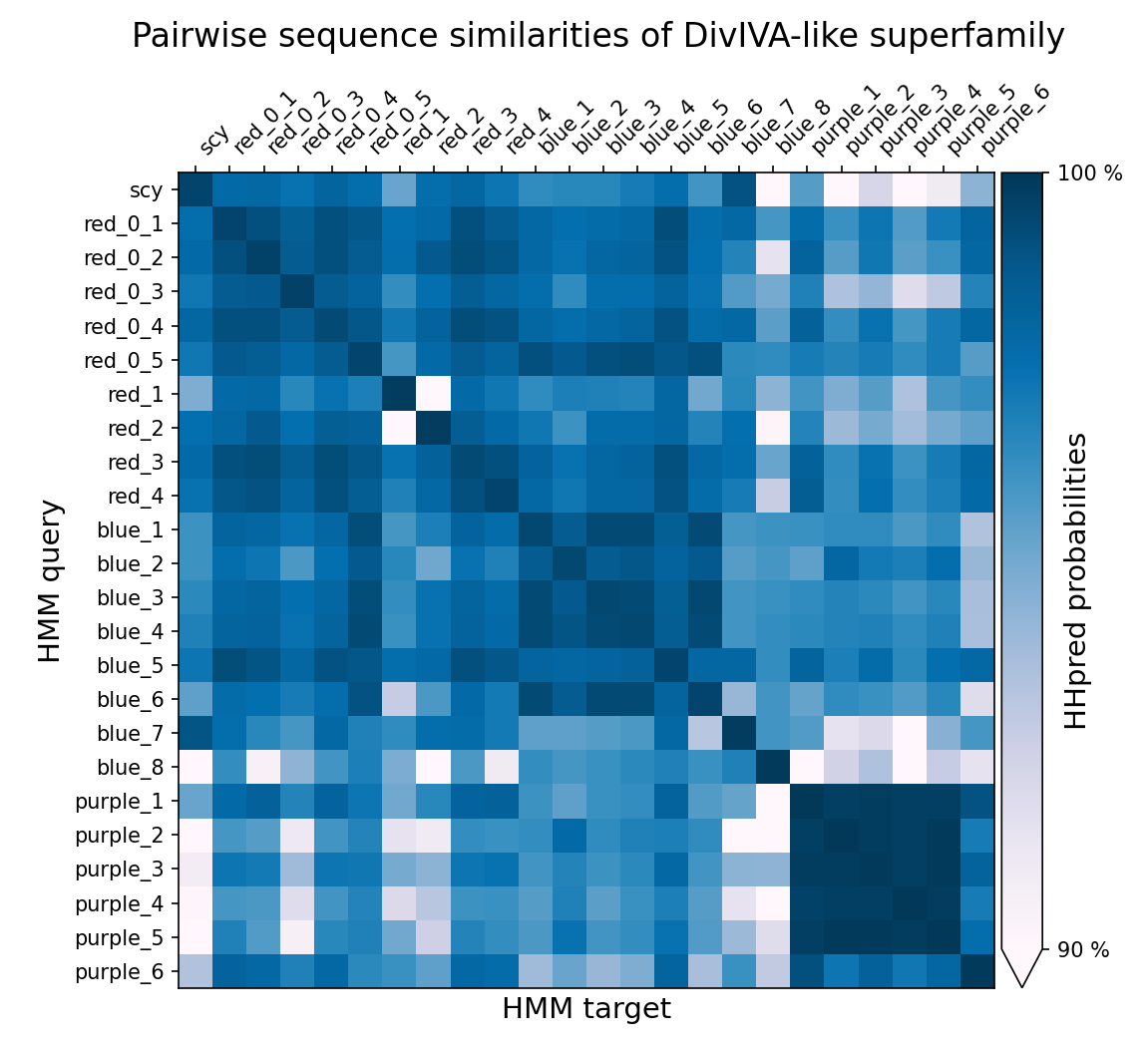
**S3 Figure. Heatmap of pairwise sequence similarities for representative members of the DivIVA-like superfamily**.

The sequences correspond to those annotated in the Mendeley data repository (<https://data.mendeley.com/datasets/bn627zbymx>). For the computation of the similarities, we only considered the DivIVA-like domains of every sequence, as defined in the main manuscript, and ran the HHpred HMM-HMM comparison tool for every pairwise comparison. For the graphing of the data, we set a lower bound of 90%, which shows that there is a strong similarity signal both within and between groups. The lowest HHred probability score was higher than 80%.
