# Supplementary material for: A conserved motif suggests a common origin for a group of proteins involved in the cell division of Gram-positive bacteria
Source: PLoS One. 2023 Jan 20;18(1):e0273136. doi: 10.1371/journal.pone.0273136 (PMC9858780; doi:10.1371/journal.pone.0273136)
Supplement: Attachment — Submitted filename: Plos Comments.doc [file pone.0273136.s002.doc]

The manuscript entitled, "**A conserved motif suggests a common origin for a group of proteins involved in the cell division of Gram-positive bacteria**", submitted by Goikoetxea M et.al. have conducted a study on proteins, involved in cell division DivIVA, GpsB, FilP, and Scy. A conserved N-terminal sequence motif has been identified, despite of their variability in occurrence & phenotypic profile. The motif, having coiled-coil helix structure is probably required for dimerization. They were collectively termed as DivIVA-like domain. The super secondary structure, consisting of two or more α-helices, wind around a central axis. The coiled coil structure, made by the repeats of seven residues, with a hydrophobic core. The hendecads repeats display similar properties. All this information has been procured by using various bioinformatics tool, like HMM-HMM, PFAM & HMMER. They MSA & BLAST of DivIVA, GpsB, FilP and Scy, and picked up homologues in other species.

However, the data obtained in this study is just obtained by computational tools, requires validation, and is not enough to accept for publication in present form. Thus, in my opinion it **CAN NOT** be accepted for publication.

**Major Points-**

1) All the information supported is not supported by experimental data. The results need experimental support.

2) There is no evolutionary tree or any figure to support the common origin of the proteins.

3) The study failed to explain how authors used GY[DN]xx[QE]V[DN] used to collect the sequence in NR3O database. How this pattern has been selected.

4) GpsB is mentioned as a paralog of DivIVA & Scy is mentioned as a paralog of FilP but no experimental proof has been mentioned to support the statement.

5) The results are not enough to identify a protein family. More experimental work is required.
