# Supplementary material for: A conserved motif suggests a common origin for a group of proteins involved in the cell division of Gram-positive bacteria
Source: PLoS One. 2023 Jan 20;18(1):e0273136. doi: 10.1371/journal.pone.0273136 (PMC9858780; doi:10.1371/journal.pone.0273136)
Supplement: Attachment — Submitted filename: Response to Reviewers.docx [file pone.0273136.s003.docx]

Dear Editor,

thank you for the opportunity to submit a revised version of our article describing the new DivIVA-like domain. We have made changes to the manuscript in response to the comments we received from you and the reviewers, and we detail these changes in the following.

Reviewer 1

This reviewer provided the strong opinion in their general comments and in major points 1, 4 and 5 that experimental evidence would be needed to define a new protein domain. We disagree with this assessment both because of the standards in the sequence annotation field and our personal experience. Regarding the definition of new protein domains, we note that all protein domain databases in common use today (PFAM, SMART, CDD) were defined on the basis of sequence comparisons alone and the domains therein more than a decade before the first structures became available. In the structure classification databases (SCOP, CATH, ECOD) as well, homology, as inferred from sequence comparisons, is also the first classification criterium. These domains were mostly defined long before their functions could be ascertained, and for most domains recognized today, even the general biological activity continues to be elusive. We ourselves have contributed more than 50 such domains over the years, which are published and have become part of the domain databases. In this article we have done nothing else than apply the standards in the field to the definition of a new domain and we are unaware of any experimental approach which could help substantiate this definition.

In major point 2, the reviewer criticises the absence of a supporting evidence for the common origin of the DivIVA-like domains in different proteins. We are surprised by this comment because we have explained in detail in the paper the statistical criteria for inclusion of proteins in our cluster map (Figure 2) and we think that the BLAST p-value cutoff of 1E-15 that we used is extremely stringent. Nevertheless, we have now included in the supplementary material a heatmap of pairwise sequence similarities for representative members of the clusters we described, and hope that the reviewer will be convinced by the extremely strong signal of similarity.

In major point 3, the reviewer criticises the abscence of an explanation for how we obtained and used the pattern characteristic for DivIVA-like proteins. The generation of the pattern in the MEME program is explained in detail in section 2 of the Methods. The use of patterns in string searches is a basic aspect of informatics so we did not considered it necessary to detail this aspect of our work. We have therefore not further addressed this point in revision.

Reviewer 2

In their main suggestion, the reviewer suggests that a phylogenetic figure summarizing the evolutionary steps proposed in our article would be a useful addition to the discussion. We agree and have included such a figure as the new Figure 4. At the level of resolution of our study, we are not aware of gene loss for any of these proteins that may have happened in specific lineages, nor of clear events of horizontal transfer superimposed on orthologous descent.

In the minor comments, the reviewer suggests potential problems with the representation of sequences in the alignment panel C versus motif panel D. We understand the points made, and agree that the way the figure legend was written was easy to misinterpret. We have therefore revised the figure legend, as well as the layout of panel C, in order to clarify the relevant points.

The reviewer also points out that some cautionary comments should be added to the paper concerning the use of AlphaFold models. We understand this point perfectly as well, all the more since we were assessors for the CASP14 experiment, and wish to point out two things in response: The first is that experimental structures are also models and have again and again produced erroneous outcomes due for example to crystal contacts, non-physiological buffer conditions, truncations needed for crystalization, and flawed model building (we note here the presence of several cis-prolines in CASP targets which were incorrectly modeled by the crystalographers and corrected after AlphaFold prediction). The second is that even for very complex CASP targets AlphaFold predictions were essential for a high-resolution model of datasets that could not otherwise be interpreted, showing that these predictions are complementary to experimental structure determination, not redundant with it. We have checked our manuscript carefully for instances that might be considered to overinterpret the meaning of the AlphaFold models, but we find that we have been quite cautious throughout.

We hope that with these changes and responses our manuscript is now fit for publication.

Sincerely,

Mikel Martinez Goikoetxea and Andrei N. Lupas
